# Effect of S-triazine Ring Substitution on the Synthesis of Organic Resorcinol-Formaldehyde Xerogels

**DOI:** 10.3390/gels6030021

**Published:** 2020-07-31

**Authors:** Martin Prostredný, Caio Ledingham, Ivan A. Principe, Abdelkarim S. M. Altoumi, Ashleigh J. Fletcher

**Affiliations:** Department of Chemical and Process Engineering, University of Strathclyde, Glasgow G1 1XJ, UK; martin.prostredny@strath.ac.uk (M.P.); caioledingham@gmail.com (C.L.); ivan.alejandro.principe@gmail.com (I.A.P.); aettomee@gmail.com (A.S.M.A.)

**Keywords:** Brunauer–Emmett–Teller (BET), Barrett–Joyner–Halenda (BJH), resorcinol, formaldehyde, ammeline, melamine, cyanuric acid, xerogel

## Abstract

Resorcinol (R) and formaldehyde (F) gel synthesis has been well-studied along with alternative reagents. We present the synthesis of formaldehyde-based xerogels using chemically similar s-triazine precursors, with comparison to traditional analogues. The substitution ranges from tri-hydroxyl to tri-amine, with an intermediate species, allowing changing chemistry to be investigated. Each molecule (X) offers different acid/base properties, known to influence gel formation, as well as differences in crosslinking potential. Varying X/F ratios were selected to recreate the stoichiometry used in RF systems, where one represented higher F to match the increased reaction sites of the additives. X/C ratios were selected to probe different catalyst (C) ratios, while working within the range likely to produce viable gels. Results obtained show little impact for ammeline as an additive due to its similarity to resorcinol (activation sites and pK_a_); while melamine and cyanuric acid show differing behavior depending on the level of addition. Low concentrations show melamine to have the most impact due to increased activation and competition for formaldehyde; while at high concentrations, cyanuric acid is shown to have the greatest impact as it creates a more acidic environment, which diminishes textural character, possibly attributable to larger clusters and/or weaker cross-linking of the system.

## 1. Introduction

There have been myriad attempts to replace the key reagents used to synthesize the first porous organic materials as reported by Pekala in 1989 [1]. Traditionally, resorcinol (R) and formaldehyde (F) are used as precursors; however, alternative reagents have been used, often in the hope of changing the chemical character and physical properties of the final materials [2,3,4,5,6,7,8,9,10]. The reaction of R and F creates activated monomers that form hydroxymethyl derivatives and undergo poly-condensation reactions to create a three-dimensional extended structure. Consequently, it is seen as crucial to retain the aromatic nature when replacing R to provide a direct comparison with traditional RF gels and focus on the effect of ring substitution. Previous works have focused on replacing R with aromatic amine species to increase the basic character of the gels; such modifications allow sorbents to be used in applications requiring high levels of interaction with acidic gases, e.g., CO_2_ or H_2_S [2,3,4], and feed into potential applications in gas separation and storage [2], improved electrochemical performance [5] or enhanced surface functionalization for reaction with biological entities [6,7,8]. The partial substitution of R for another species allows for the retention of advantageous textural properties for RF-derived gels, whilst tuning the surface interactions of these materials. While these studies have looked at the effect of ring substitution and impact on the final properties of the gels [3,9,10], the role of substituted groups should also be considered.

Previous work has focused on substitution of R with melamine, and subsequent study of the impact of experimental conditions and the amount of additives on gel properties [2,3,5,9,10]. We have previously explored the substitution of R with an aromatic amine species under acid conditions and the extent to which they can be successfully incorporated into the final gel [11], with particular focus on the retention of the gas adsorption capacity. In our current work, we focus on the effect of substitution on the s-triazine ring, using a weak base as the catalyst, thus allowing for the investigation of the subtle differences that arise from the introduction of specific functional groups, and their impact of final gel textural properties.

In order to obtain porous materials, water contained within the hydrogel pores, which results from the gelation and curing steps, is usually replaced by a solvent with a lower surface tension and, preferably, lower boiling point. Introducing this step mitigates pore collapse, which would, in turn, cause material shrinkage. Acetone is a common solvent used for the solvent exchange of organic porous gels, due to its favorable properties, and as such was also utilized in this work. In traditional gel processing, a drying step would typically follow solvent exchange; this can be performed either at supercritical conditions, most commonly using supercritical CO_2_ to yield aerogels [1], or under more economical subcritical conditions, often under vacuum. Xerogels, obtained using subcritical conditions [12], are often reported in the literature, with a wide range of physical and chemical properties of the final gels [13,14,15]; therefore, an analogous processing method was used in this work to allow for comparison with such materials.

In creating hydrogels, the substitution of ring carbons with heteroatoms, and the substitution of functional moieties attached to these rings has the potential to alter the gel structures in tandem with changes in other reaction conditions, such as the R to F molar ratio (R/F), R to catalyst (C) molar ratio (R/C), solids content and initial pH of the reaction mixture. Controlled variation of many of these parameters is already known to alter the end materials, hence, keeping key parameters fixed, while varying additive nature and concentration should allow the role of these functional groups, within the synthetic process, to be determined. 

**Scheme 1:**
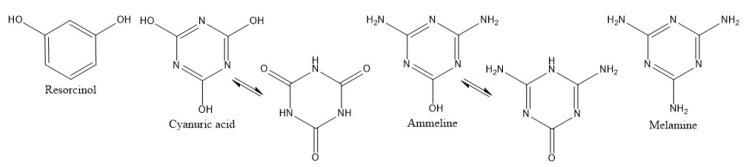
Molecular structures of Resorcinol (R), substituted s-triazine structures: Cyanuric acid (CA), Ammeline (A), Melamine (M) and their tautomers.

This paper reports a systematic study of the substitution of R in the traditional RF synthetic process to create several suites of xerogels with a range of final properties. The species selected to substitute R were ammeline (A), melamine (M) and cyanuric acid (CA), each offering the same ring heteroatom substitution while varying the functional groups present within the synthetic system, and allowing the effect of these groups on the final cross-linking of the three-dimensional product to be evaluated (Scheme 1). It is worth noting that CA is known to exist predominantly in the keto form, the tautomer shown in Scheme 1, rather than the enol form [16,17]. Resulting materials were characterized with respect to textural properties, providing insight into material differences and available surface areas for subsequent functionalization and reaction.

## 2. Results and Discussion

### 2.1. Gel Synthesis Parameters

#### 2.1.1. X/C Ratio and Initial pH

As has been traditionally undertaken in a number of RF studies, the catalyst route used here is in the basic regime. The catalyst type and amount have been shown to affect the surface area, density, porosity and mechanical characteristics of final RF-gels [14]; in this study, sodium carbonate was consistently used as the catalyst for the reaction. In such base-mediated systems, the reaction proceeds by deprotonation of R and the s-triazine species to form anions, which react with the carbon atom of F to give hydroxymethyl derivatives—these, in turn, are deprotonated and react further to form stable methylene- and ether-linked gel networks [18,19]. Increasing R/C, i.e., lower catalyst concentrations, can result in longer gelation times as fewer nucleation sites are available for cluster growth, thereby creating larger particle sizes and decreasing the overall surface area but increasing mesopore volume [20]. Conversely, decreasing R/C, i.e., higher catalyst concentrations, creates smaller, highly branched clusters that exhibit little growth prior to gelation, resulting in smaller particles and an increase in overall and micropore surface area [14]; such systems tend to better withstand the mechanical forces experienced during drying [15]. This behavior is mirrored in the observations made with respect to initial pH values, where higher values, which would result from the lower R/C ratios, demonstrate formation of smaller clusters and enhanced surface areas and vice versa [21]. The pH values around neutral to mildly acidic (5.45 to 7.35) have been shown to create gels with a mix of porous characters [22], consequently X/C ratios of 50 and 200, where X is the aromatic species, have been selected within this study. This gives a good comparison of low and high catalyst concentrations, while avoiding such high values that gelation may be problematic, both for gel formation and subsequent shrinkage on drying [23].

The pK_a_ values obtained for the four cyclic components are shown in Table 1, and indicate that both A and M have equivalent pK_a_ values (~9) to R and, hence, would be expected to demonstrate similar dissociative behavior, resulting in similar levels of acidity. By contrast, the pK_a_ value for CA is significantly lower than the other three molecules (at 6.5), meaning that the resulting synthetic matrix will exhibit a lower initial pH, which will affect the subsequent gelation processes. Within the reactions performed in this study, it was evident that the addition of M and A resulted in little change in the initial pH compared to the counterparts created using R only. In both cases, increasing amounts of the additive had minor effects on the overall pH, which was consistently >7. In the case of CA, the initial pH was always below 7, falling as low as 6.22 for the gel created using 20 wt% of CA.

#### 2.1.2. X/F Ratio

The stoichiometric ratio for the synthesis of RF gels is understood to be 0.5. The majority of studies utilize this; however, there are a handful that have included other R/F ratios in their studies, such as 0.25 in an attempt to control the pore character of the final RF gel [27]. Here, X/F = 0.5 was used as it offers the stoichiometric proportions for aromatic species (X) reacting with F; in addition, X/F = 0.25 offers an increased amount of F, which may be required for reaction, particularly with M and A, as these have the ability for reaction at a greater number of sites on the molecule. It should be noted that previous work has reported that increasing F may cause a dilution effect, which results in an increase in particle size and collapse of the mesoporous structure of the material; however, too little F can limit derivative formation and hinder gelation [28], hence, additional F has been provided but still to a limited degree.

The suite of gels obtained allows for the effect of the X/C ratio and the impact of varying F concentration in tandem with additive replacement in the traditional RF matrix. In each case, gels obtained using R and F only for synthesis conditions of R/F = 0.25 or 0.5 combined with R/C ratios of 50 and 200 are included to show the direct effect of the additive to the final gel.

### 2.2. Gels Synthesized Using S-Triazine Additives

#### 2.2.1. 1 wt% Additive Substitution

**Figure 1:**
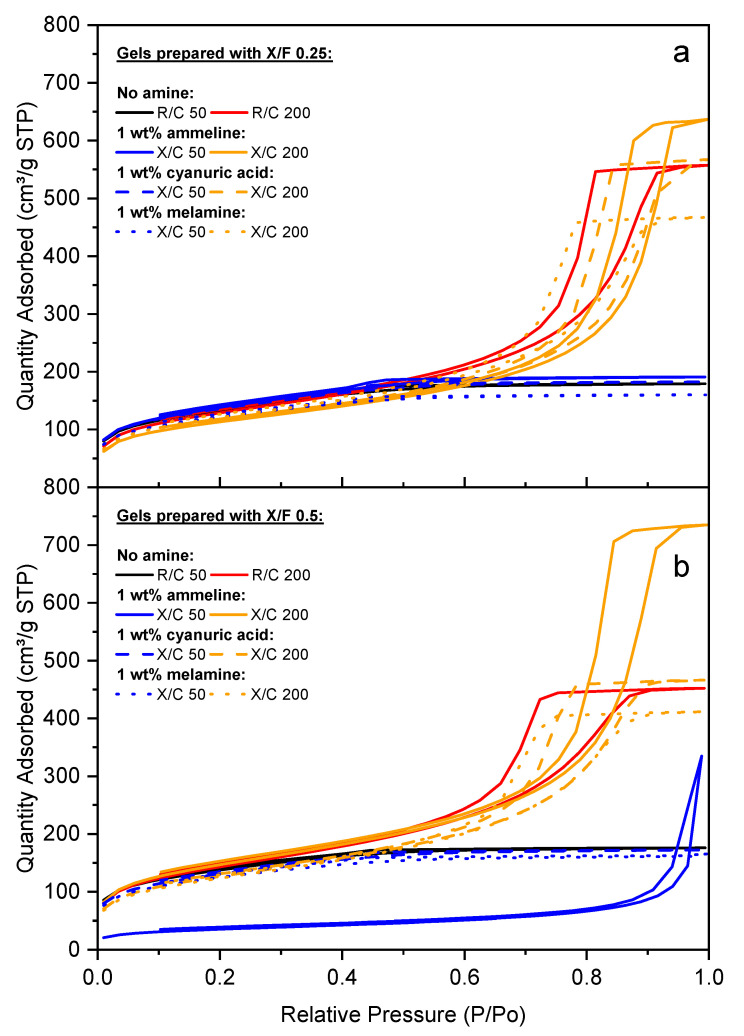
Nitrogen sorption isotherms for gels prepared with 1 wt% of additives and with (**a**) X/F 0.25 and (**b**) X/F 0.5.

**Figure 2:**
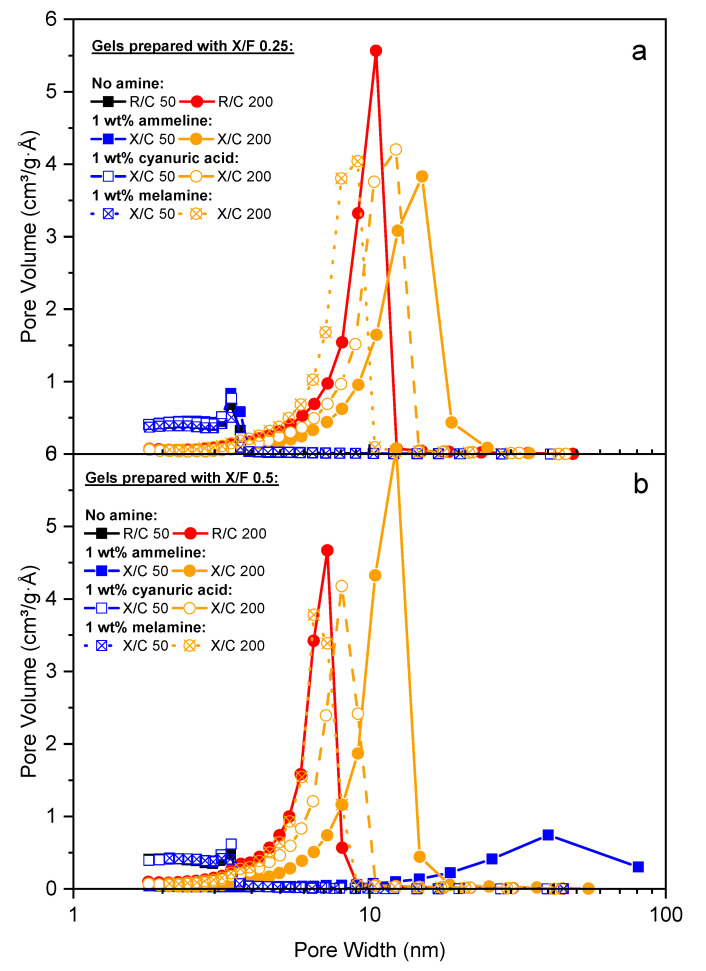
Pore size distributions for gels prepared with 1 wt% of additives with (**a**) X/F 0.25 and (**b**) X/F 0.5.

In the case of 1 wt% additive substitution, it is clear that there is some difference for the gels obtained using A (Figure 1 and Figure 2). For X/F = 0.25 there is little impact, noticeably only for A, with a minor change in the pore size distribution (Figure 2a), indicating that, for both X/C ratios, with the presence of sufficient F, additives have a minimal impact on the final gel. This difference is more prevalent for the X/F = 0.5 system, where the isotherms for A demonstrate a shift in the hysteresis loops towards higher relative pressures (Figure 1b), which is then reflected in the larger average pore size observed for these gels (Figure 2b). The impact seems to be independent of X/C ratio; however, the pore size distributions for X/C 50 appear to more consistent across the four gel types, which may be a consequence of the higher concentration of catalyst available within the synthetic matrix, hence, less competition between the two species in each reaction make-up.

#### 2.2.2. 5 wt% Additive Substitution

**Figure 3:**
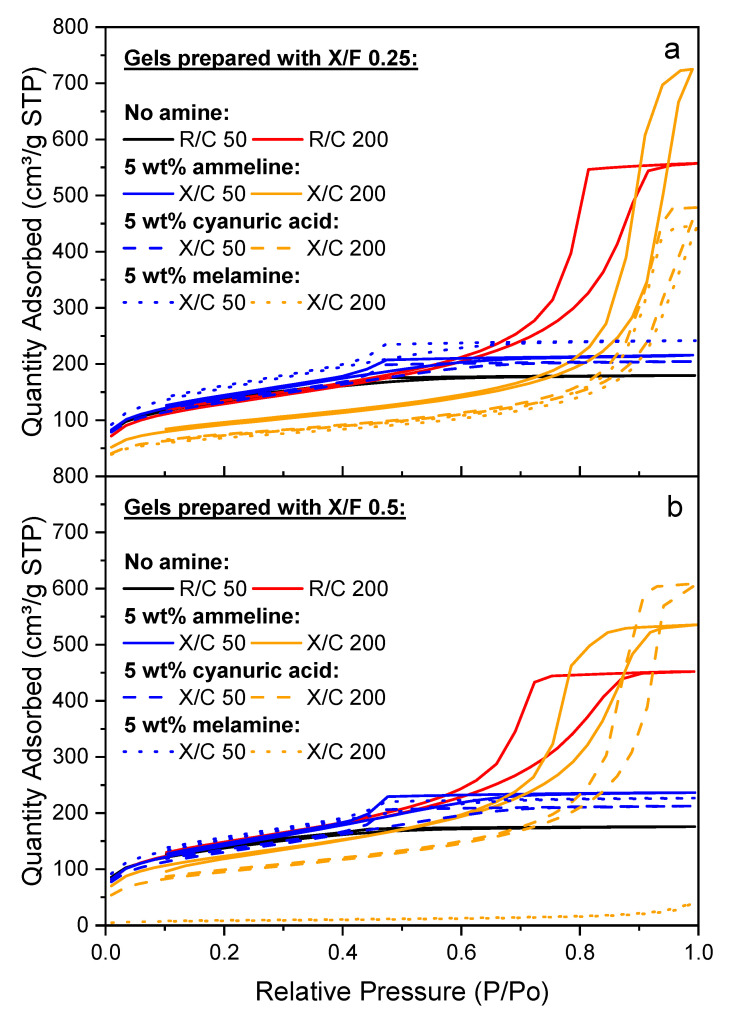
Nitrogen sorption isotherms for gels prepared with 5 wt% of additives and with (**a**) X/F 0.25 and (**b**) X/F 0.5.

**Figure 4:**
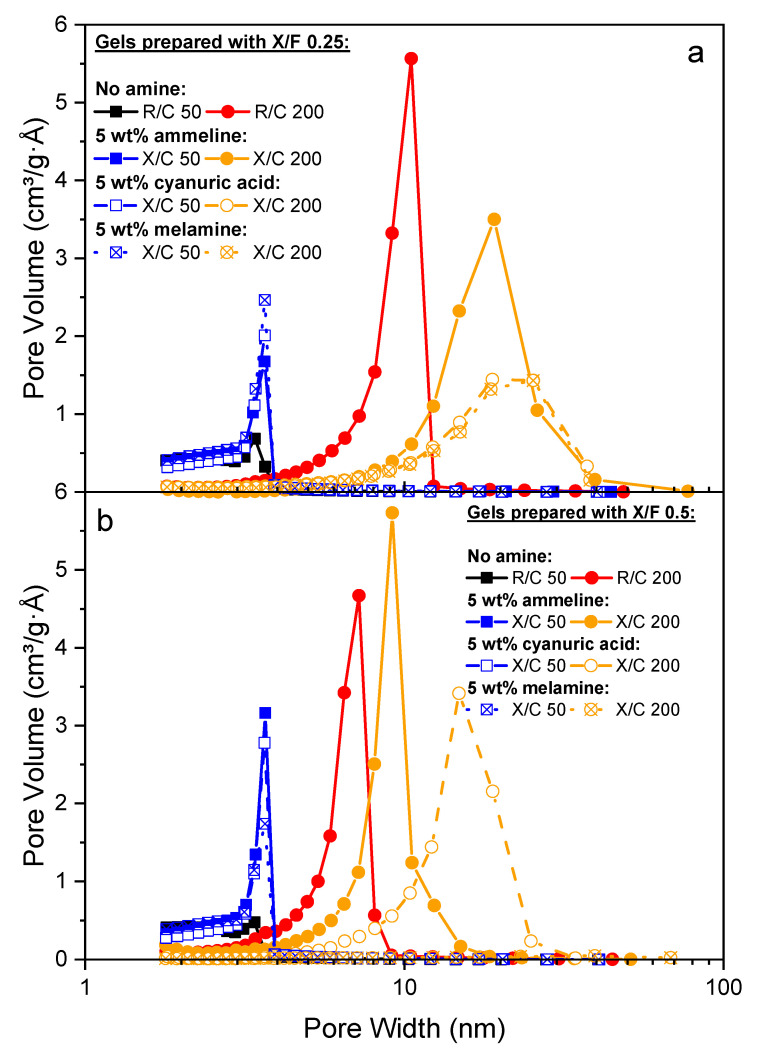
Pore size distributions for gels prepared with 5 wt% of additives with (**a**) X/F 0.25 and (**b**) X/F 0.5.

Increasing the level of substitution to 5 wt% of a given additive is similar to the lower level of 1 wt% for X/C 50 at both X/F ratios (Figure 3 and Figure 4). However, it is evident that the gels synthesized at X/C 200 demonstrate significant effects on the final porous structure upon adulteration of the synthetic matrix. The differences are less marked for X/F = 0.25, with a minor shift in the isotherms for all three s-triazines (Figure 3a) and very little difference in their comparative pore size distributions, which are all shifted to the same average pore diameter (Figure 4a). This is similar to the 1 wt% systems and may likely be due to the increased amount of F available for reaction compared with X/C 200, where the three s-triazines show markedly different impacts. The addition of A has the least impact, for similar initial pH (see Section 2.1.1), and the minor difference in the available active sites on the ring molecule (three for R and four for A) shows a small shift in the isotherm (Figure 3b) and corresponding pore size distribution (Figure 4b) as A primarily has little effect on the final gel textural properties.

By contrast, CA, which offers a lower pK_a_ [25], resulting in a lower initial pH, is affected by the more acidic conditions and the gel is, consequently, less stable; likely producing larger clusters, which shifts the isotherm hysteresis loop to higher relative pressures and increases the average pore diameter. Finally, the addition of M has the greatest impact on the gel obtained for 5 wt% addition, with a significant decrease in uptake obtained for the material and an absence of pores within the micro- and meso-pore regions; such character may limit the potential of these materials for some applications, including gas separation or aqueous phase adsorption, where these channels would typically provide enhanced adsorption capacity or transportation of media. The difference in impact for M when comparing the X/F ratios is related to the availability of F within the reaction matrix; as M has a maximum of six reactive sites, compared with three for R, there is greater competition between R and M for F, resulting in a decrease in activated R, which reduces cross-linking and produces a weaker, less porous gel. Within the 5 wt% suite of materials, it is evident that the order of similarity to RF gels is R > A > CA > M, suggesting that the impact of competition for F, which is enhanced for M, is more significant than the pH adjustment offered by addition of CA.

#### 2.2.3. 10 wt% Additive Substitution

**Figure 5:**
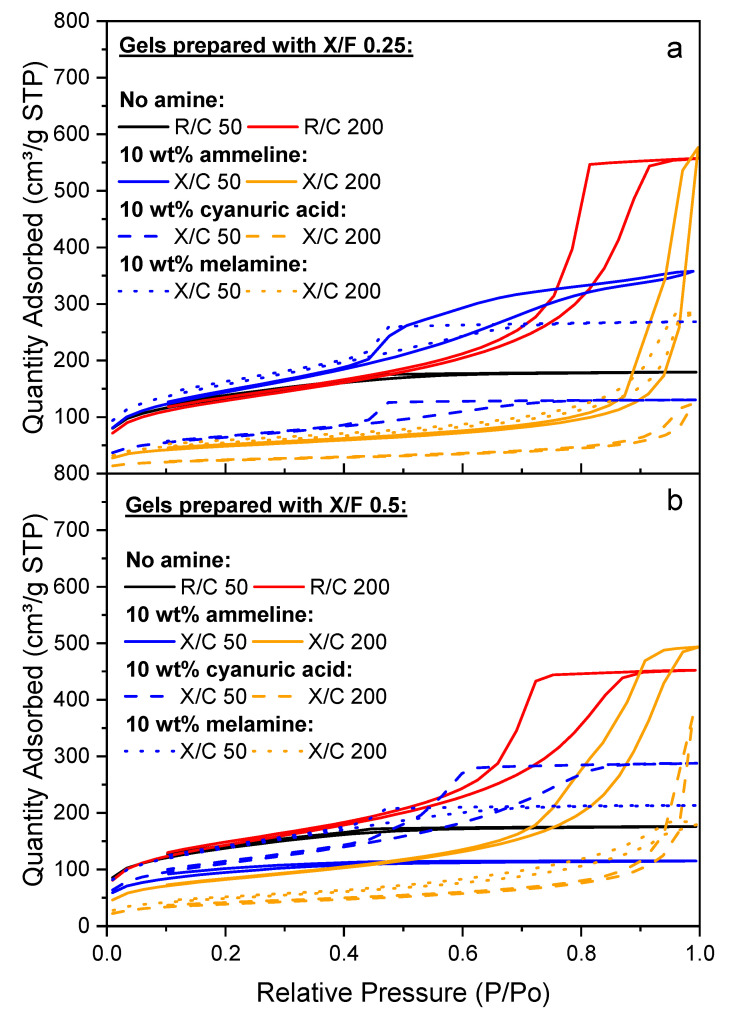
Nitrogen sorption isotherms for gels prepared with 10 wt% of additives and with (**a**) X/F 0.25 and (**b**) X/F 0.5.

**Figure 6:**
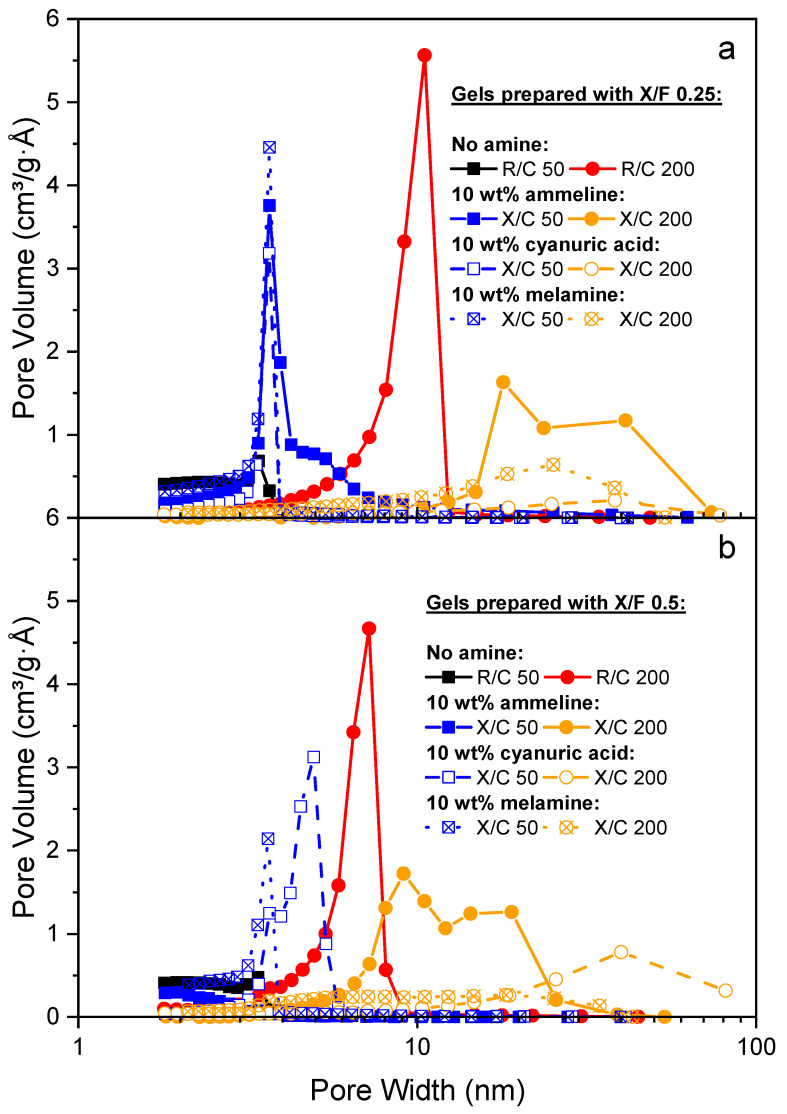
Pore size distributions for gels prepared with 10 wt% of additives with (**a**) X/F 0.25 and (**b**) X/F 0.5.

The results obtained for 10 wt% addition of s-triazine species (Figure 5 and Figure 6), show similar trends to those obtained at 5 wt% in that A shows the least impact, with CA next and M demonstrating the greatest impact. For X/F = 0.25, the pore diameters are shifted to higher values in all cases (Figure 6a), but, in line with previous observations, the three additives show similar results in terms of average pore dimeter, suggesting that again, with sufficient F available, the difference in additive species is consistent irrespective of the chemistry of the added species. However, as for previous additive levels, it is evident that at X/F = 0.5, where less F is available for reaction, the addition of M has a significant impact on the final gel at X/C 50, while both M and CA have significant detrimental impact at X/C 200. This suggests that the additional reactive sites of M compete with R at both concentrations of the catalyst and it is the ratio of X/F that more greatly affects the final textural properties of the gels. At X/C 50 and X/F = 0.5, CA shows some greater impact at 10 wt% addition than for 5 wt%, which may be a result of the more acidic conditions created within the reaction (initial pH ~6.6 compared with ~6.8 for 5 wt%); this increases cluster size before gelation occurs, reducing the surface area (comparing 495 m^2^ g^−1^ for the RF gel with 468 m^2^ g^−1^ for 5 wt% CA and 396 m^2^ g^−1^ for 10 wt%, see Appendix A) and overall pore size. At the higher X/C ratio, CA also exhibits a greater impact on the final gel, which can be ascribed to a combination of the more acidic conditions created by adding the increased amount of CA, and the lower amount of catalyst available, which reduces the number of nucleation sites, increasing average cluster size and reducing surface area and average pore size (Figure 6b).

#### 2.2.4. 15 wt% Additive Substitution

**Figure 7:**
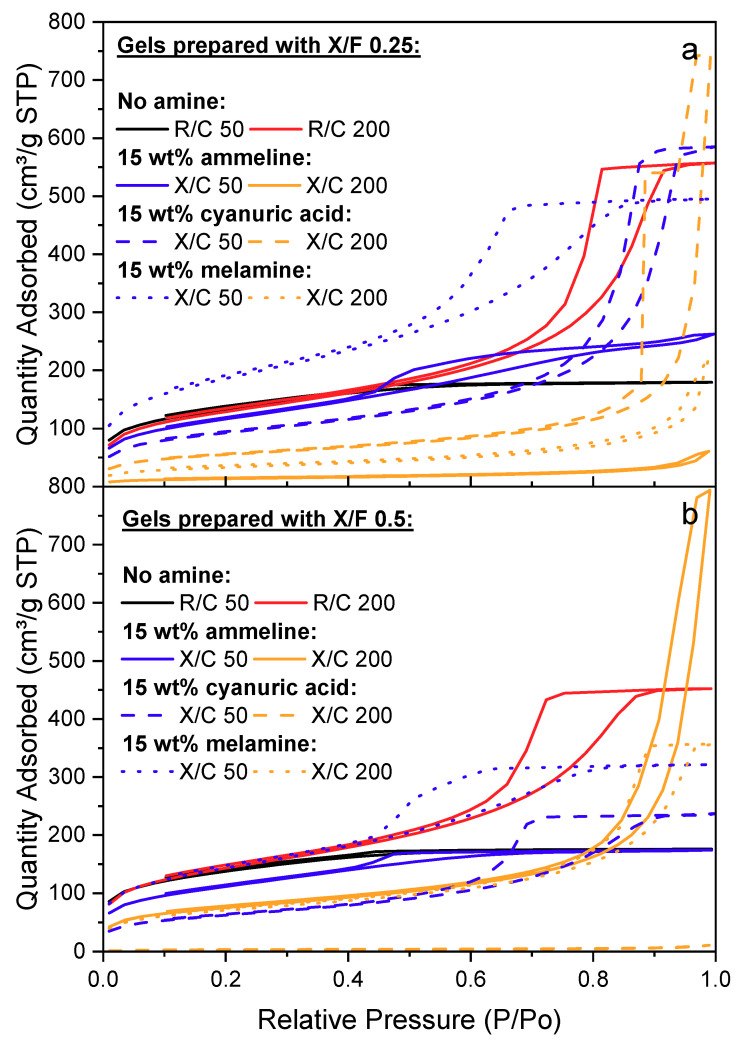
Nitrogen sorption isotherms for gels prepared with 15 wt% of additives and with (**a**) X/F 0.25 and (**b**) X/F 0.5.

**Figure 8:**
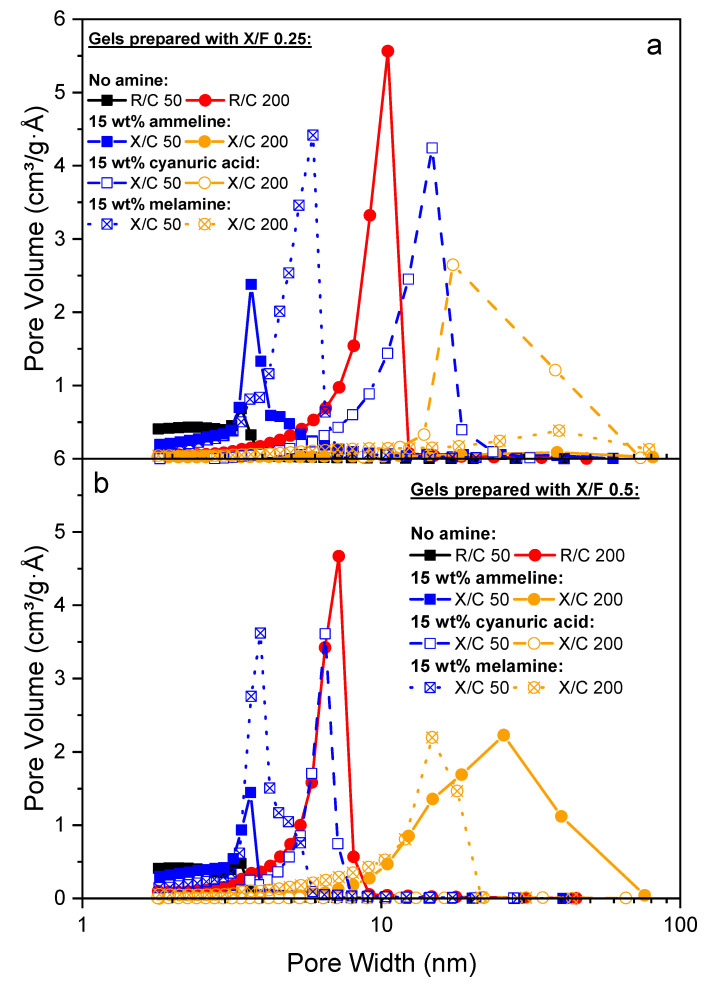
Pore size distributions for gels prepared with 15 wt% of additives with (**a**) X/F 0.25 and (**b**) X/F 0.5.

Addition of 15 wt% results in significantly different behavior to the three previous addition levels (Figure 7 and Figure 8). At X/C 50, the higher level of F offered by X/F = 0.25 assists in the creation of stable gels with greater pore volume, shifted to slightly higher average pore diameters for A and M. While both molecules have similar pK_a_ values to R [24,25,26], they show contrasting results for the X/F = 0.5 systems, where A demonstrates little impact on the trends obtained for R, while M shows slightly more impact with the reduced availability of F. The X/C 200 results show much more impact for substation of R, most notably at X/F = 0.5, where the more acidic CA now exceeds the impact shown by M, which has the least impact on pH, as the influence of the increasing amount of the acid (reducing the pH to ~6.4) has a greater impact on the final materials than competition for F. Contrastingly, the pH for M increases from ~7.2 at low amounts to ~7.4 for 15 wt%, which helps stabilize M with respect to R, reducing its competitiveness for F; the enhanced textural properties can be ascribed to the higher cross-linking potential for M, which must occur in these samples. Again, as for all previous systems, A shows the least impact on the final gel properties; having a higher pK_a_ it activates less easily than R, so is less competitive for F and the final gel is very similar to an RF analogue in all cases. Now, the trend of similarity to RF gels is R > A > M > CA, suggesting that the impact of increasing acidity for CA gels is more significant than the competition for F, which is reduced as the activation of M decreases. 

#### 2.2.5. 20 wt% Additive Substitution

**Figure 9:**
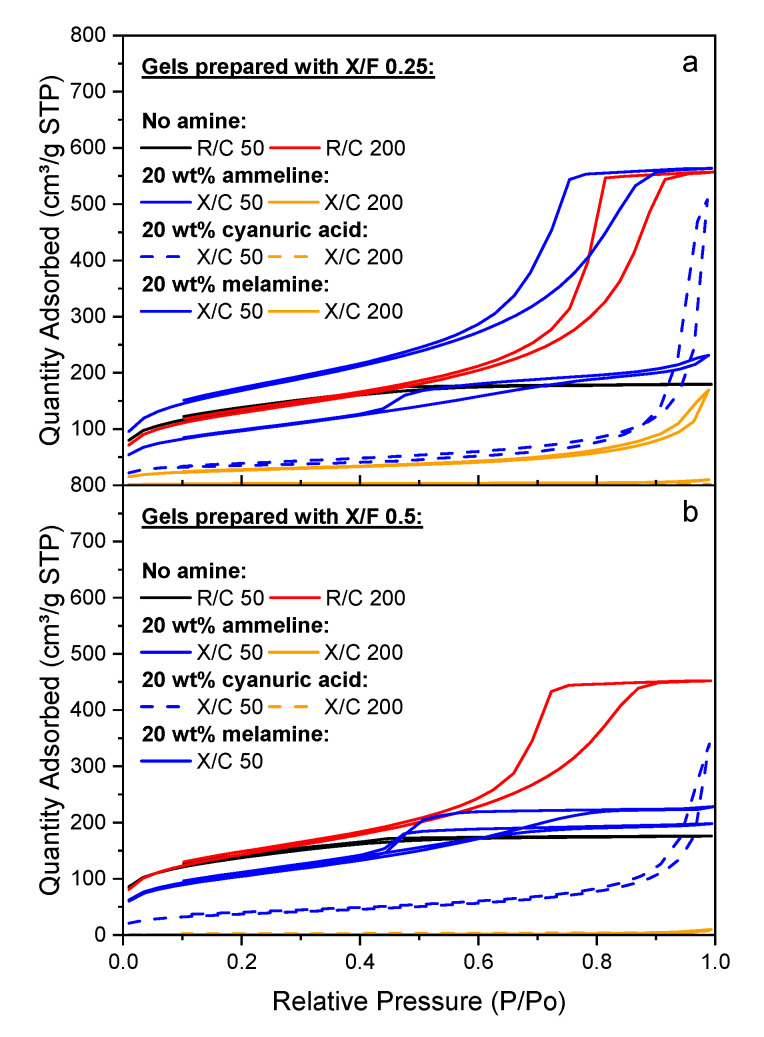
Nitrogen sorption isotherms for gels prepared with 20 wt% of additives and with (**a**) X/F 0.25 and (**b**) X/F 0.5.

**Figure 10:**
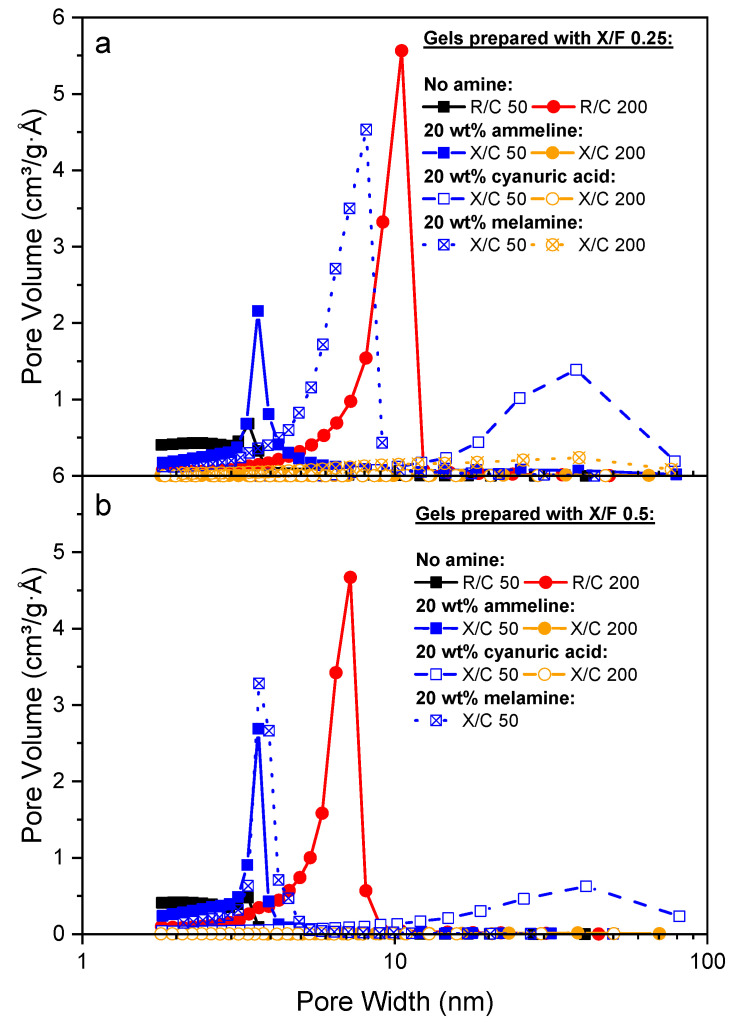
Pore size distributions for gels prepared with 20 wt% of additives with (**a**) X/F 0.25 and (**b**) X/F 0.5.

The final weight percentage tested was 20 wt%, above which gels were not attempted as it was already becoming evident that the increasing level of additive was either being detrimental to the final gel or preventing gelation from occurring at all, which was the case for M with X/F = 0.5 and X/C 200 (Figure 9b and Figure 10b). From visual observation, it was evident that the final structure of the material was weak, suggestive of a lower level of cross-linking, and so feeble that it was unable to undergo the required solvent exchange and drying processes. From the results shown in Figure 9b and Figure 10b, it is evident that the gels obtained for 20 wt% of A and CA using X/F = 0.5 and X/C 200 were also weak materials with negligible adsorption capacity and no evident micro- or meso-porosity. Gels for the same X/C but greater levels of F available (i.e., X/F = 0.25) fared little better with melamine producing the only alternative gel with some adsorption potential, likely due to its similar pK_a_. For X/C 50, there is the continued trend that A creates the most similar gel to RF, with M next and CA exhibiting the greatest impact (Figure 9a and Figure 10a); this order can again be ascribed to the increased acidity of the reaction mixture as the amount of CA is increased, while the other two species, with their more basic nature, show little influence. As for 15 wt%, the greater cross-linking potential from M is likely to enhance the gel structure obtained and produces the increased adsorption capacities observed in Figure 9a and Figure 9b. The X/C 50 gels benefit from the greater concentration of catalyst, which helps retain the gel structure, even at X/F = 0.5; however, CA still creates a very weak structure with substantially wider pores than the three other gels.

## 3. Conclusions

The gels obtained in this study provide insight into the impact of replacing resorcinol (R) in the traditional RF gel synthesis. Using specific wt% amounts of ammeline (A), cyanuric acid (CA) and melamine (M), it is possible to alter the final textural properties; in most cases where a change is observed, this results in an increase in average pore diameter. This behavior results from a range of influences, including (i) pH moderation, which is particularly impactful for CA, which has a lower pK_a_ than all other molecules used, (ii) the potential of each molecule to form cross-links in the polycondensation step, which is greatest for M and (iii) the number of possible reactive sites on each molecule (three for A and R, four for CA and six for M). It is evident that there is a subtle interplay between the chemistry of the molecule, in terms of its potential for activation and cross-linking, and the effect that adding each species has on the process parameters, including pH. The results obtained here have potential to inform the tailored synthesis of RF gels and systems with additives, which can be especially useful in designing bespoke materials including sorbents.

## 4. Materials and Methods 

### 4.1. Gel Formation

All gel samples were prepared using a procedure analogous to a previously developed procedure for RF gels containing aromatic amine substitutes [11]. Resorcinol (R, Sigma Aldrich, Gillingham, UK, Reagent Plus, 99%), melamine (M, Sigma Aldrich, 99%), ammeline (A, TCI, Oxford, UK, ≥95%) and cyanuric acid (CA, Sigma Aldrich, 98%) were used as aromatic reagents in this work and formaldehyde (F) was used in form of formalin solution (Sigma Aldrich, 37wt% F in water, containing 10–15 wt% methanol as a polymerization inhibitor). In order to promote reactions between the aromatic species and formaldehyde, sodium carbonate (C, Sigma Aldrich, anhydrous, ≥99.5%) was used to adjust the reaction mixture pH and is referred to, in this work, as catalyst. All chemicals were used as received from the supplying company as detailed for each component, and deionized water was prepared in-house (Millpore Elix® 5 with Progard® 2, Merck, Watford, UK). 

All reaction solutions were prepared using 20 w/v% solids content, taking into account all aromatic reagents (R, M, A, CA), sodium carbonate, and F. The total liquid volume used, unless otherwise stated, was 30 cm^3^, made up of water and methanol, which is contributed by the formalin solution used as a source of F. The ratios of catalyst to aromatic species were reported on a molar basis (X/C), with values of 50 and 200. Similarly, the ratio of aromatic (X) and F (i.e., X/F) was set on a molar basis and X/F values of 0.25 and 0.5 were used in this work. The concentrations of aromatic additives were selected in this work as 1, 5, 10, 15 and 20 wt%, and a set of RF gels without any additive, prepared at the same conditions, was used for comparison. Due to lower solubility of the amine species in the reaction mixture, these were dissolved in a glass jar with a magnetic stirrer in a premeasured volume of deionized water along with the corresponding amount of R, heating up the solution to ~60 °C and allowing it to cool down prior to addition of the catalyst and formalin solution. After the reaction mixture was left stirring for 30 min, the stirrer bar was removed from the solution and pH of the solution was measured using pH20 bench top pH meter fitted with a HI 1110-B pH probe (Hanna Instruments, Leighton Buzzard, UK). 

Subsequently, the jar lids were hand-tightened, before transferring into an oven (Memmert UFE400, Schwabach, Germany), pre-heated to 85 °C. In order to allow sufficient time for gelation and curing of the samples, in line with our previous work [11,29], the samples were left in the oven for 3 days. After the gelation period, the jars were taken out of the oven and allowed to cool to ambient temperature.

In order to replace water contained within the gel structure to reduce potential structure collapse and material shrinkage, caused by high surface tension of water, a solvent exchange step was performed using acetone (Sigma Aldrich, ≥99.5%). The gels were cut into smaller pieces (~1 cm) prior to washing them with acetone to remove excess water from sample and jar surfaces. The initial amount of acetone was drained and 90 cm^3^ of fresh acetone was added to the jar. Afterwards, the lids were replaced and wrapped in paraffin film to minimize acetone losses during the solvent exchange period. The sealed jars were put on a shaker unit (VWR 3500 Analog Orbital Shaker, Lutterworth, UK,), to promote acetone mixing, for 3 days. If a sample was deemed too soft, as determined during the cutting step, the jar was left on a bench top, and periodically mixed gently, preventing mechanical damage to the gel structure. 

Finally, after the solvent exchange step, the acetone was drained and the jars with samples were moved to vacuum oven (Towson and Mercer 1425 Digital Vacuum Oven, Stretford, UK). The drying temperature was set to match the gelation temperature, in order to avoid any structural changes due to thermal effects. After 2 days of drying under vacuum, the resulting xerogel samples were transferred to labelled sample tubes for storage.

### 4.2. Textural Characterisation

The prepared gels were analyzed using nitrogen sorption measurements in order to determine their textural properties. Nitrogen sorption was carried out at −196 °C using a Micromeritics ASAP 2420 surface area and porosity analyzer. In order to remove any previously adsorbed species on the sample surface, a degas process was followed, involving outgassing under vacuum below 10 µmHg at 50 °C for 30 min and then at 110 °C for 2 h. For analysis, a 40 pressure point adsorption and 30 pressure point desorption cycle was used. In the cases where the total surface area of the analyzed sample was less than 100 m^2^, a volume displacement insert was used to reduce measurement errors, as per the guidelines of the instrument manufacturer. All samples were analyzed for surface area [m^2^/g] using Brunauer–Emmett–Teller (BET) theory [30] applying the Rouquerol correction [31] for microporous samples; total pore volume [cm^3^/g]; micropore volume [cm^3^/g] using the t-plot method [32]; and average pore size [nm] from the Barrett–Joyner–Halenda method [33].

## Figures and Tables

**Table 1:** pK_a_ values for resorcinol and s-triazine species used in this study.

Species	pK_a_
Resorcinol	pK_a1_ 9.20 [24]; pK_a2_ 10.9 [24]
Cyanuric acid	6.5 [25]
Ammeline	9.65 [26]
Melamine	8.95 [25]

## Appendix A

**Table A1:** Textural properties and initial reaction mixture pH values for RF gels prepared without any additive.

R/C Ratio	R/F Ratio	S_BET_ [m^2^/g]	V_µ_ [cm^3^/g]	V_T_ [cm^3^/g]	$\bar{\varphi }$ [nm]	pH
50	0.25	480	0.08	0.28	3	7.5
	0.5	495	0.11	0.27	3	7.4
200	0.25	465	0.05	0.86	8	7.0
	0.5	514	0.05	0.70	6	7.1

S_BET_—surface area from BET analysis; V_T_—total pore volume determined from adsorption at p/p_o_ ~1; V_µ_—micropore volume determined using t-plot method; $\bar{\varphi }$—average pore width from BJH analysis. Errors are omitted from the table as all values are reported to an accuracy less than the largest error for each variable.

**Table A2:** Textural properties and initial reaction mixture pH values for gels with ammeline as the additive.

[A] wt%	X/C Ratio	X/F Ratio	S_BET_ [m^2^/g]	V_µ_ [cm^3^/g]	V_T_ [cm^3^/g]	$\bar{\varphi }$ [nm]	pH
1	50	0.25	497	0.08	0.30	3	7.5
		0.5	129	0.02	0.52	24	7.2
	200	0.25	408	0.05	0.99	11	6.9
		0.5	530	0.06	1.14	10	7.0
5	50	0.25	503	0.05	0.33	3	7.5
		0.5	507	0.04	0.37	3	7.5
	200	0.25	331	0.04	1.12	16	6.8
		0.5	444	0.05	0.83	8	7.1
10	50	0.25	517	0.03	0.55	4	7.4
		0.5	342	0.10	0.18	2	7.7
	200	0.25	174	0.02	0.89	26	6.9
		0.5	295	0.03	0.76	11	7.1
15	50	0.25	417	0.03	0.41	4	7.5
		0.5	400	0.04	0.27	3	7.5
	200	0.25	48	0.01	0.09	11	6.8
		0.5	270	0.03	1.23	21	7.1
20	50	0.25	349	0.02	0.36	4	7.4
		0.5	391	0.02	0.31	3	7.4
	200	0.25	8	-	0.02	12	6.8
		0.5	3	-	0.02	17	7.0

S_BET_—surface area from BET analysis; V_T_—total pore volume determined from adsorption at p/p_o_ ~1; V_µ_—micropore volume determined using t-plot method; $\bar{\varphi }$—average pore width from BJH analysis. Errors are omitted from the table as all values are reported to an accuracy less than the largest error for each variable.

**Table A3:** Textural properties and initial reaction mixture pH values for gels with cyanuric acid as the additive.

[CA] wt%	X/C Ratio	X/F Ratio	S_BET_ [m^2^/g]	V_µ_ [cm^3^/g]	V_T_ [cm^3^/g]	$\bar{\varphi }$ [nm]	pH
1	50	0.25	488	0.08	0.28	3	7.1
		0.5	469	0.09	0.27	3	7.0
	200	0.25	411	0.04	0.88	9	6.7
		0.5	455	0.05	0.72	7	7.1
5	50	0.25	474	0.05	0.32	3	7.0
		0.5	468	0.05	0.33	3	6.9
	200	0.25	262	0.03	0.74	14	6.3
		0.5	344	0.04	0.94	13	7.0
10	50	0.25	231	0.01	0.20	3	6.8
		0.5	396	0.03	0.44	4	6.9
	200	0.25	85	0.01	0.19	12	6.0
		0.5	139	0.02	0.58	23	6.8
15	50	0.25	333	0.03	0.90	12	6.3
		0.5	223	0.02	0.37	6	6.7
	200	0.25	199	0.03	1.15	21	6.3
		0.5	9	-	0.02	10	6.3
20	50	0.25	140	0.02	0.79	29	6.4
		0.5	133	0.02	0.53	20	6.5
	200	0.25	1	-	-	19	5.7
		0.5	5	-	0.01	10	6.4

S_BET_—surface area from BET analysis; V_T_—total pore volume determined from adsorption at p/p_o_ ~1; V_µ_—micropore volume determined using t-plot method; $\bar{\varphi }$—average pore width from BJH analysis. Errors are omitted from the table as all values are reported to an accuracy less than the largest error for each variable.

**Table A4:** Textural properties and initial reaction mixture pH values for gels with melamine as the additive.

[M] wt%	X/C Ratio	X/F Ratio	S_BET_ [m^2^/g]	V_µ_ [cm^3^/g]	V_T_ [cm^3^/g]	$\bar{\varphi }$ [nm]	pH
1	50	0.25	439	0.08	0.25	3	7.2
		0.5	509	0.03	0.50	3	-
	200	0.25	436	0.04	0.72	7	-
		0.5	445	0.04	0.64	6	-
5	50	0.25	562	0.06	0.37	3	7.3
		0.5	547	0.07	0.35	3	-
	200	0.25	247	0.03	0.69	14	7.1
		0.5	29	0.00	0.06	12	6.5
10	50	0.25	564	0.06	0.42	3	7.2
		0.5	447	0.08	0.26	3	-
	200	0.25	194	0.03	0.44	12	-
		0.5	176	0.02	0.28	7	-
15	50	0.25	668	0.06	0.77	5	7.4
		0.5	514	0.04	0.50	4	7.7
	200	0.25	119	0.02	0.34	15	-
		0.5	254	0.03	0.55	10	6.7
20	50	0.25	606	0.06	0.87	6	7.6
		0.5	376	0.03	0.35	4	-
	200	0.25	96	0.01	0.26	13	-
		0.5	-	-	-	-	-

S_BET_—surface area from BET analysis; V_T_—total pore volume determined from adsorption at p/p_o_ ~1; V_µ_—micropore volume determined using t-plot method; $\bar{\varphi }$—average pore width from BJH analysis. Errors are omitted from the table as all values are reported to an accuracy less than the largest error for each variable.
